# Basilar artery fenestration in migraine patient

**DOI:** 10.1002/ccr3.9107

**Published:** 2024-06-21

**Authors:** Aneesh Rahangdale, Kateryna Kurako

**Affiliations:** ^1^ Departments of Psychiatry and Neurology HCA Florida Capital Hospital Tallahassee Florida USA; ^2^ Southeast Neurology Specialists Tallahassee Florida USA

**Keywords:** basilar artery fenestration, magnetic resonance angiography, migraine, MRA

## Abstract

Basilar artery fenestration should be considered in migraine patients, especially with a cerebrovascular family history, necessitating annual magnetic resonance imaging/angiography (MRI/MRA) monitoring and careful assessment of birth control regimens to mitigate risks.

## 1 INTRODUCTION

Basilar artery fenestration is a rare vascular anomaly characterized by the division of the basilar artery into two parallel channels that subsequently rejoin that often remains asymptomatic and incidentally discovered during imaging studies for unrelated conditions.^1^, ^2^ However, it can occasionally be associated with neurological symptoms such as headaches, vertigo, or transient ischemic attacks due to altered hemodynamics or microemboli within the duplicated segment. The incidence of basilar artery fenestration has been reported to range from 0.28% to 6% based on autopsy studies, and 0.02% to 2.07% based on angiographic studies.^1^, ^2^, ^3^ Fenestration of the basilar artery is most commonly found in the proximal portion near the vertebrobasilar junction, while fenestration of the middle and distal portions is less common.^1^, ^2^

Basilar artery fenestration has been associated with various vascular pathologies, including aneurysms, arteriovenous malformations, and ischemic events.^1^, ^2^, ^3^ The formation of aneurysms at the site of basilar artery fenestration is a well‐recognized complication, with an incidence ranging from 15% to 34%.^2^, ^3^ The complex geometric structure of the fenestration, its proximity to vital structures, and the challenging nature of the region for surgical access increase the risks of morbidity and mortality associated with the treatment of these aneurysms.^2^ Accurate diagnosis through advanced imaging modalities such as magnetic resonance imaging/angiography (MRI/MRA) and digital subtraction angiography (DSA) is essential for appropriate management and prognostication. Differential diagnoses may include vascular variants such as basilar artery dolichoectasia or aneurysms, necessitating thorough diagnostic evaluation.

The management of basilar artery fenestration depends on the clinical presentation, associated symptoms, and imaging findings. Long‐term follow‐up is crucial to monitor for any changes in clinical status or potential complications associated with this vascular anomaly. Asymptomatic cases typically require conservative management with close observation, while symptomatic cases may warrant further interventions such as antiplatelet therapy, surgical clipping, or endovascular procedures. Endovascular treatment has become the preferred approach for managing basilar artery fenestration aneurysms, as it offers a less invasive alternative to open surgical techniques.^2^, ^3^ Various endovascular techniques have been employed, including coil embolization, stent‐assisted coiling, and flow diversion.^2^, ^3^ Studies have reported high technical success rates and favorable outcomes with endovascular management of these lesions.^2^, ^3^

However, the rarity of basilar artery fenestration aneurysms has limited the available literature, and experience with different endovascular treatment modalities remains relatively limited.^2^, ^3^ Ongoing research and case reports continue to expand the understanding of the optimal management strategies for these complex vascular anomalies.

## 2 CASE PRESENTATION

A 35‐year‐old female presented with complaints of frequent migraines, occurring 10–12 days per month. The patient reported that her headaches typically began with a throbbing sensation in the left frontal region, often accompanied by nausea, sensitivity to light and sound, and occasionally vomiting. These symptoms usually escalated over a period of 30 min to an hour and could last between 4 and 6 h. There were no signs of sudden onset or “thunderclap” headaches suggestive of aneurysm rupture or blood leakage. The headaches were exacerbated by weather changes, strong smells, and menstrual cycles. She had been tried on various medications for migraine management without significant improvement. Her medical history was notable for recently starting an etonogestrel/ethinyl estradiol vaginal ring for birth control. Family history was significant for maternal great‐grandmother and grandmother passing away from a ruptured brain aneurysm. Physical examination was unremarkable apart from non‐focal neurological findings.

### 2.1 Tried meds

Topiramate, gabapentin, sumatriptan, eletriptan, ubrogepant, and aspirin/paracetamol/caffeine.

### 2.2 Review of systems

Frequent headaches, otherwise unremarkable (no loss of consciousness, no weakness, no numbness, no seizures, no dizziness, no tremor, no gait dysfunction, and no paralysis).

### 2.3 Physical examination

Within normal limits, non‐focal, no signs of neck stiffness or meningeal irritation.

### 2.4 Diagnostic assessment

**FIGURE 1:**
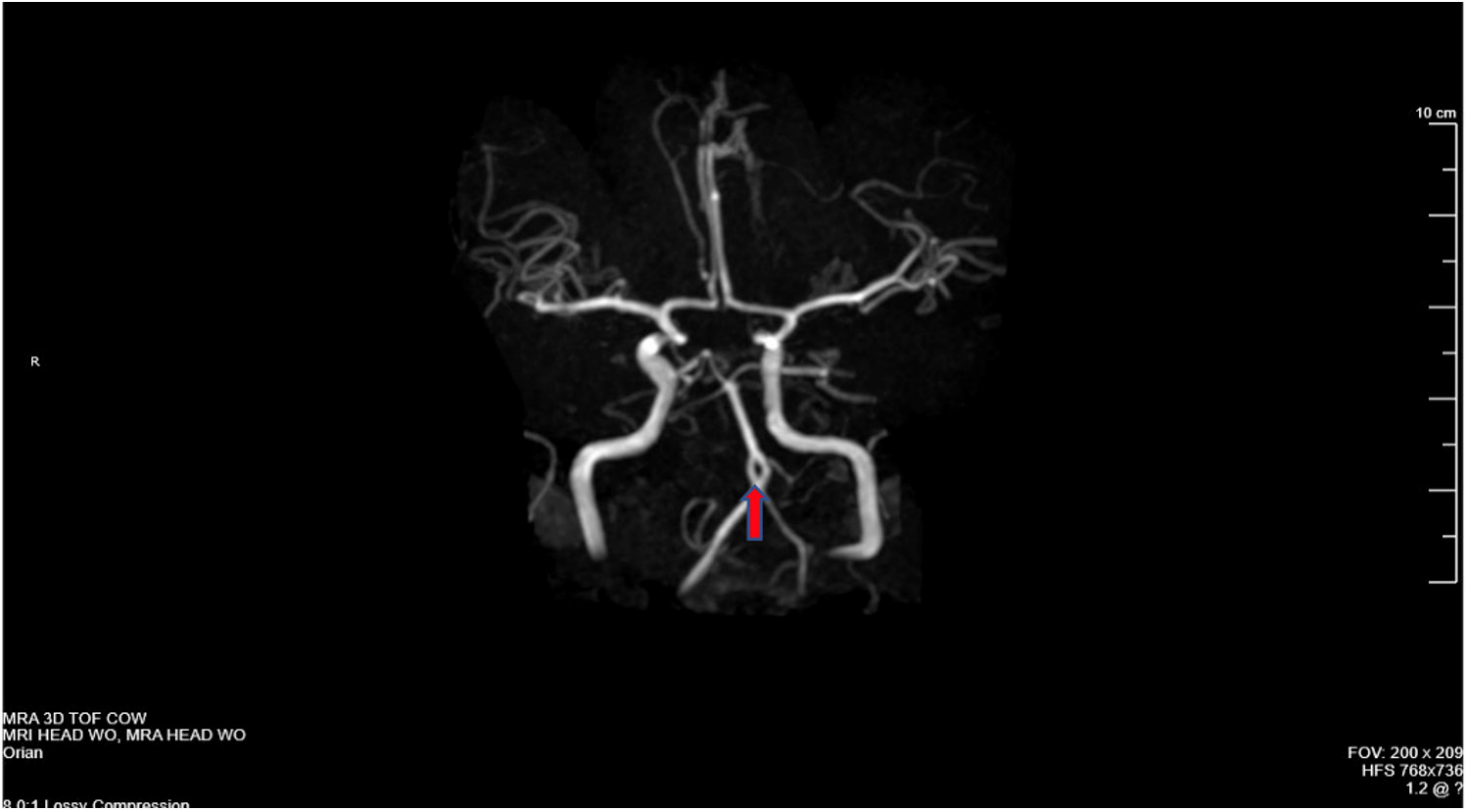
Magnetic resonance angiography (MRA) Head without contrast highlights basilar artery fenestration measuring 6 mm in craniocaudal diameter. The red arrow is pointing to the anatomic finding. No other intracranial abnormalities were noted. Magnetic resonance imaging/angiography (MRI/MRA) revealed the characteristic features of basilar artery fenestration, with two parallel channels of the basilar artery separated by a septum, rejoining distally to form a single basilar artery. No evidence of thrombosis, aneurysm, or other associated vascular abnormalities was noted.

Magnetic resonance imaging/angiography (MRI/MRA) of the head and neck revealed a proximal basilar artery fenestration measuring approximately 6 mm in craniocaudal diameter, as seen in Figure 1. No other intracranial abnormalities were noted. The patient's migraines were likely multifactorial, with the fenestration potentially contributing to altered hemodynamics and triggering factors.

### 2.5 Management and outcome

The patient was advised to discontinue her etonogestrel/ethinyl estradiol vaginal ring due to the increased risk of clots and strokes with migraines. She was given rimegepant samples for abortive migraine care and galcanezumab injection for preventive treatment. Lifestyle modifications and healthy practices were encouraged. Follow‐up magnetic resonance imaging/angiography (MRI/MRA) was recommended annually to monitor the basilar artery fenestration. Over a follow‐up period of 6 months, the patient remained stable without progression of symptoms or new neurological deficits.

## 3 DISCUSSION

Basilar artery fenestration is a rare vascular variant that may predispose individuals to aneurysm formation. In patients with migraines, especially those with a family history of aneurysms, thorough evaluation including imaging studies is crucial. Management involves a combination of pharmacotherapy, lifestyle modifications, and regular monitoring to mitigate potential complications.

This case underscores the importance of considering and assessing for vascular anomalies like basilar artery fenestration with magnetic resonance angiography (MRA) in patients with migraines, especially when there is a family history of cerebrovascular disorders. Moreover, clinicians ought to evaluate birth control regimen of migraine patients, especially whether it is estrogen‐containing birth control. The patient's use of an estrogen‐containing vaginal ring is significant given the known impact of hormonal fluctuations on migraine pathophysiology. Estrogen can influence vascular reactivity and central nervous system processing, potentially exacerbating migraine attacks. Considering her family history of cerebrovascular disorders, it was recommended to discontinue the etonogestrel/ethinyl estradiol vaginal ring to mitigate the increased risk of thrombotic events and hormone‐related migraine exacerbations. Monitoring patients with basilar artery fenestration with annual magnetic resonance imaging/angiography (MRI/MRA) seems like a reasonable plan balancing risks and benefits. In the meantime, we recommend appropriate blood pressure control and lifestyle modifications to lower the risk of aneurysm formation and/or rupture. Nonetheless, we recommend having a low threshold for referral to vascular surgery if anatomical findings are changing.

## AUTHOR CONTRIBUTIONS

**Aneesh Rahangdale:** Conceptualization; data curation; formal analysis; investigation; methodology; project administration; resources; software; validation; visualization; writing – original draft; writing – review and editing. **Kateryna Kurako:** Project administration; supervision; writing – review and editing.

## FUNDING INFORMATION

This study was not supported by any sponsor or funder.

## CONFLICT OF INTEREST STATEMENT

The authors have no conflicts of interest to declare.

## ETHICS STATEMENT

This study protocol was reviewed and approved HCA Healthcare PUBCLEAR, approval number MS #2486. This research was supported in whole or in part by HCA Healthcare and/or an HCA Healthcare affiliated entity. The views expressed in this publication represent those of the authors and do not necessarily represent the official views of HCA Healthcare or any of its affiliated entities.

## CONSENT

Written informed consent was obtained from the patient to publish this report in accordance with the journal's patient consent policy.

## Data Availability

All data generated or analyzed during this study are included in this article. Further enquiries can be directed to the corresponding author. The data that support the findings of this study are not publicly available due to their containing information that could compromise the privacy of research participants but are available from the corresponding author [AR] upon reasonable request.
